# Sensitive detection of pathway perturbations in cancers

**DOI:** 10.1186/1471-2105-13-S3-S9

**Published:** 2012-03-21

**Authors:** Corban G Rivera, Brett M Tyler, TM Murali

**Affiliations:** 1 Department of Computer Science, Virginia Tech, Blacksburg, VA, USA; 2 Virginia Bioinformatics Institute, Virginia Tech, Blacksburg, VA, USA; 3 ICTAS Center for Systems Biology of Engineered Tissues, Virginia Tech, Blacksburg, VA, USA; 4 Department of Biomedical Engineering, Johns Hopkins University, Baltimore, MD 21218, USA

## Abstract

**Background:**

The normal functioning of a living cell is characterized by complex interaction networks involving many different types of molecules. Associations detected between diseases and perturbations in well-defined pathways within such interaction networks have the potential to illuminate the molecular mechanisms underlying disease progression and response to treatment.

**Results:**

In this paper, we present a computational method that compares expression profiles of genes in cancer samples to samples from normal tissues in order to detect perturbations of pre-defined pathways in the cancer. In contrast to many previous methods, our scoring function approach explicitly takes into account the interactions between the gene products in a pathway. Moreover, we compute the sub-pathway that has the highest score, as opposed to merely computing the score for the entire pathway. We use a permutation test to assess the statistical significance of the most perturbed sub-pathway. We apply our method to 20 pathways in the Netpath database and to the Global Cancer Map of gene expression in 18 cancers. We demonstrate that our method yields more sensitive results than alternatives that do not consider interactions or measure the perturbation of a pathway as a whole. We perform a sensitivity analysis to show that our approach is robust to modest changes in the input data. Our method confirms numerous well-known connections between pathways and cancers.

**Conclusions:**

Our results indicate that integrating differential gene expression with the interaction structure in a pathway is a powerful approach for detecting links between a cancer and the pathways perturbed in it. Our results also suggest that even well-studied pathways may be perturbed only partially in any given cancer. Further analysis of cancer-specific sub-pathways may shed new light on the similarities and differences between cancers.

## Introduction

Complex diseases such as cancer are associated with the alteration or dis-regulation of multiple pathways and processes in the cell. Discovering and cataloging which pathways are perturbed in each type of cancer is important for improving our understanding of the mechanisms underlying these diseases. In particular, such studies can pin-point pathways that may be uniquely perturbed in one or a small number of related cancers, thus providing potential targets for therapeutic studies.

Many methods have been developed to study the activation of pre-defined gene sets in human diseases and tissues [1-7]. In this context, a "gene set" is usually taken to be a collection of genes that share a common attribute, e.g., Gene Ontology annotation or membership in a pathway. For instance, Subramanian et al. [3] developed "Gene Set Enrichment Analysis" to test if a gene set is differentially expressed in two phenotypes by ranking all genes by some measure (say, the *t *statistic) and using a modified Kolmogorov-Smirnov statistic to decide whether the genes in the set have surprisingly high or low ranks. Segal et al. [8] used a hierarchical clustering algorithm to combine pre-defined gene sets into modules. They characterized gene-expression profiles in specific (sets of) tumors as a combination of activated and de-activated modules.

These methods ignore physical or functional interactions between the genes (or their products) in a gene set. Analysis of gene expression measurements in the context of the interaction structure inherent in a pathway can take into account both perturbations in gene expression and the topological properties of the network. More recent methods have sought to capture information about the activation of a pathway from the perspective of the interactions in it. A number of these techniques, reviewed by [9], have been developed for case-control data, for which we can compute *p*-values reflecting the statistical significance of the differential expression of each gene between the samples in the treatment and those in the control [10-14]. Draghici et al. [10] combined a term that captured the significance of the genes in a pathway with an additional weighted term that measured how well the data matches the expected pattern of induction and repression, as encoded by the interactions in the pathway. Efroni et al. [11] used pathway perturbation measurements to predict prognosis and tumor grade. Both approaches measure the perturbation of a pathway in its entirety. Thus, they may not be sensitive to situations when only a sub-pathway is highly perturbed.

Related techniques analyze gene expression measurements made under an experimental condition in the context of a large-scale protein-protein interaction network (often integrated from multiple sources) in order to determine the sub-network of interactions that respond to the experimental condition [15-19]. These approaches have primarily been used for determining the global response network perturbed in the cell in a particular condition, especially since most experimentally-determined protein interactions have not yet been explicitly associated with pathways.

### Our contributions

In this paper, we develop a systematic methodology to detect which pathways are perturbed in a disease. Here, we use the term *pathway *to refer to a network of physical interactions between genes and gene products that together perform a specific biological function. Given the interactions in a pathway (e.g., the TNF alpha pathway) and genome-wide case-control gene expression data, i.e., measurements for a disease phenotype (e.g., melanoma) and a control phenotype (e.g., normal skin cells), our method computes the sub-pathway that is most perturbed in the disease (when compared to the control). Thus, our method combines the features of the two classes of methods discussed above: (i) it treats a pathway as a network of interconnected molecules rather than merely as a set of genes and gene products; (ii) it is sensitive to the possibility that the pathway is not perturbed in its entirety but that only some portion of it is significantly perturbed; and (iii) it can be applied to specific, well-defined pathways that a scientist may be interested in studying.

Our algorithm takes the interactions in a pathway *P *and case-control gene expression measurements as input. We first assess the differential expression of each gene in *P*. We develop a statistic based on the Liptak-Stouffer *z*-score that measures the combined perturbation of the genes in *P*. This statistic takes into account both the interactions in *P *and the differential expression of each gene. We use this statistic to compute which sub-pathway of *P *is maximally perturbed. Finally, we use a permutation-based test to assess the statistical significance of the maximally-perturbed sub-pathway.

### Our results

We applied this approach to 20 cancer and immune signaling pathways in the Netpath database [20]. We used gene expression measurements in the Global Cancer Map (GCM) [21]. The GCM dataset spans 18 cancers and 13 normal tissues. First, we showed that the scores of perturbed sub-pathways computed by our method are much more statistically significant than the scores of the complete pathways. Second, we compared our results to those obtained by applying three techniques that analyze case-control gene expression data: ActiveModules [16], Gene Set Enrichment Analysis (GSEA) [3], and Sub-GSE [7]. ActiveModules integrates the gene expression data with protein interaction networks in order to find highly perturbed sub-networks. GSEA and Sub-GSE are network-free approaches that find gene sets that are highly perturbed in the gene expression data. Our method showed much better sensitivity than both ActiveModules and GSEA in detecting perturbed sub-pathways. The comparison between our approach and Sub-GSE was mixed. Third, our method was robust to missing data, specifically to the removal of gene expression samples from the input. Finally, we found ample literature support for a number of pathway-cancer associations detected by our approach. Taken together, these results underscore the importance of carefully incorporating pathway structure into the analysis of gene expression data. We considered other recent approaches for comparison, which use mutual information to score individual genes [15], measure the synergistic relationship among a set of genes [22], or use biclustering to account for phenotypic variation among individuals [23,24]. However, the number of samples per cancer in the GCM dataset is not sufficient to support robust computation of mutual information. These counts may not large enough to yield informative biclusters either. Therefore, we decided not to compare these methods with our approach in this paper.

## Algorithms

We describe our approach in three stages. First, we formalize a measure of how perturbed a sub-network of a pathway is in a case-control gene expression data set. Next, we describe how to compute a sub-network that maximizes this measure. Finally, we discuss how we measure the statistical significance of the most perturbed sub-network.

### Condition-specific pathway activation

We define a *pathway P *= (*G*, *I*) to be a graph composed of a set *G *of genes and a set *I *of physical or functional interactions between the genes in *G *or their gene products. Typically, *P *may be composed of multiple connected components. Given genome-wide gene expression measurements in multiple patients diagnosed with a disease in a tissue and from normal samples of that tissue, our goal is to determine whether the pathway *P *= (*G*, *I*) is perturbed in the disease (when compared to normal tissue) and to compute the subgraph of *P *that is most perturbed in the disease.

For each gene *g *∈ *G*, let *p*(*g*) denote the *p*-value of its differential expression in the disease (when compared to normal tissue). We computed *p*(*g*) as the *p*-value of the two-sided *t*-test under the null hypothesis that the distributions of the expression values of *g *in the disease samples and in the normal samples have identical means (but may have different variances). We note that our pathway perturbation algorithm can take as input any gene expression pre-processing method that computes *p*-values for differential gene expression. We converted the *p*-value into a z-score *z*(*g*) = *N^-^*^1^(1 - *p*(*g*)), where *N^-^*^1 ^is the inverse of the normal cumulative distribution function [16]. At this stage, we did not impose a cut-off on *z*(*g*). Instead, we included all genes in subsequent analysis. The rationale for this choice was that while individual genes may not be differentially expressed to a statistically-significant extent, significant perturbations may be noticeable at the level of sets of genes [25].

The method we developed takes the interaction structure of *P *into account. Let *Q *= (*G'*, *I'*) be a subgraph of *P*. We define the *degree d_Q_*(*g*) of a gene *g *∈ *I' *to be the number of interactions in *I' *that are incident on *g*. We define the *perturbation *of a subgraph *Q*(*G'*, *I'*) of *P *to be the weighted Liptak-Stouffer z-score [26]

$$z\left(Q\right)=\frac{\sum_{g\in G^{\prime }}d_{Q}\left(g\right)z\left(g\right)}{\sqrt{\sum_{g\in G^{\prime }}d_{Q}^{2}\left(g\right)}}.$$

The numerator of *z*(*Q*) is the weighted sum of the z-scores of all genes that appear in *Q*, where each gene is weighted by the number of interactions in *Q *that are incident on it. Dividing by the square root of the sum of squared gene degrees ensures that *z*(*Q*) is normally distributed with mean 0 and standard deviation 1, under the assumption that the *z*-scores for the individual genes arise from a normal distribution. Thus, this formulation of perturbation combines *p*-values over multiple genes in a statistically-sound way [27]. Each gene in *Q *contributes both its z-score and its degree in *Q *to *z*(*Q*). Thus, *z*(*Q*) incorporates both the differential expression of the genes in *Q *as well as the network of interactions between them.

### Computing the sub-pathway that is most perturbed

Among all subgraphs of *P*, let $\hat{P}$ be the one with maximum value of perturbation. Since $\hat{P}$ is the most differentially-perturbed subgraph of *P*, we use its perturbation to assess the overall perturbation of *P*. Thus, our formulation does not require that every gene in *P *be differentially expressed in order for us to declare that *P *itself is perturbed in the disease. We now describe how we compute $\hat{P}$. Note that we do not require that $\hat{P}$ be connected, since *P *itself may not be connected. Ideker et al. demonstrated that a similar problem is NP-complete [16]. Hence, we use a heuristic approach based on simulated annealing. Although simulated annealing is a very well known technique, we describe it below and sketch it in Algorithm 1 for the sake of completeness. To initialize $\hat{P}$, we include each interaction in *P *with a uniform probability of 0.5.

We perform the following series of operations for 100*|I| *iterations. (Recall that *I *is the set of interactions in *P*.) We select a node or an edge uniformly at random from *P*. Let the selected element be *a*. If *a *is already in $\hat{P}$, we delete it from $\hat{P}$; if *a *is a node, we also delete all edges that are incident on *a *from $\hat{P}$. If *a *is not a member of $\hat{P}$, we add it to $\hat{P}$; if *a *is a node, we insert into $\hat{P}$ all edges that were incident on *a *in *P*. Let ${\hat{P}}^{\prime }$ be the resulting subgraph. We compute *z*(${\hat{P}}^{\prime }$) and compare it to *z*($\hat{P}$). If *z*(${\hat{P}}^{\prime }$) is larger, we accept the modification, since we have increased the z-score. Otherwise, we accept the modification with a probability of $e^{\left(z{\left(\hat{P}\right)}^{\prime }-z\left(\hat{P}\right)\right)/T}$, where *T *is the temperature in the current iteration. Over the iterations, we decrease the temperature *T *geometrically from *T_s _*= 100 to *T_e _*= 10^-5^. We output the final value of $\hat{P}$.

**Algorithm 1 **Compute $\hat{P}$, the subgraph of *P *with the maximum perturbation.

Initialize $\hat{P}$ by including each interaction in *P *with probability 0.5.

*T *← *T_s_*

**for ***i *= 1 *.. *. 100*|I| ***do**

   ${\hat{P}}^{\prime }\leftarrow \hat{P}$

   Select a node or an edge *a *∈ *P *uniformly at random.

   **if ***a *is in $\hat{P}$** then**

      Delete *a *from ${\hat{P}}^{\prime }$.

   **else**

      Insert *a *into ${\hat{P}}^{\prime }$.

   **end if**

   **if ***z*(${\hat{P}}^{\prime }$) *> z*($\hat{P}$) **then**

      Set $\hat{P}$ to be ${\hat{P}}^{\prime }$

   **else**

      Set $\hat{P}$ to be ${\hat{P}}^{\prime }$ with probability $e^{\left(z\left({\hat{P}}^{\prime }\right)-z\left(\hat{P}\right)\right)/T}$

   **end if**

   $T\leftarrow T\times e^{\frac{\text{log}\left(\frac{T_{e}}{T_{s}}\right)}{100|I|}}$

end for

#### Remarks

We experimented with other options within this framework such as starting with an empty subgraph and performing more than 100*|I| *iterations. We did not find a significant benefit from either of these choices, i.e., the score of most perturbed sub-pathway did not increase substantially (data not shown). We also found that including the addition and deletion of nodes (along with their incident edges) yielded subgraphs with much larger scores than those obtained by addition and deletion of edges alone.

#### Estimating the statistical significance of perturbed pathways

A potential drawback of our definition of *z*($\hat{P}$) is that it assumes that the z-scores of the individual genes are independent. To ensure that *z*($\hat{P}$) was not an over estimate of the significance of a perturbed pathway as a result of this assumption, we performed a permutation-based test to compute an empirical estimate of statistical significance. To build a null distribution for a disease and pathway *P*, we repeated the following procedure *k *times, where *k *varies depending on the analysis performed (see "Results" for the values we used):

(i) We permuted node labels (and associated gene expression data) in the pathway. Let *k *be the number of genes in *P*. We replaced these *k *genes with *k *other genes, selected uniformly at random from a universe of genes (defined below). Let $\tilde{P}$ be the new pathway. Note that $\tilde{P}$ and *P *are isomorphic to each other, i.e., they have identical interaction structures.

(ii) We obtained the *z*-scores of the genes in $\tilde{P}$ from the gene expression data set for the disease.

(iii) We used the simulated annealing algorithm to compute *z*($\tilde{P}$).

In the first step, we defined the universe to be the intersection of the set of all genes measured in the gene expression data set for the disease and the set of genes whose products were present in a protein interaction network containing 9352 proteins and 39890 interactions (assembled from multiple sources [28-31]). We used these two sets so that every gene in $\tilde{P}$ (i) would have gene expression values and (ii) had a protein product that was known to participate in at least one interaction.

We computed the p-value for *z*($\hat{P}$) as the fraction of random trials where *z*($\tilde{P}$) *> z*($\hat{P}$). Since we tested multiple pathway-disease pairs, we controlled the false discovery rate using the method of Benjamini and Hochberg [32]. We used the adjusted *p*-value in all of the subsequent analysis.

## Results

After describing the pathway and gene expression datasets we used, we present our results in five stages. First, we evaluate whether the most-perturbed pathway we computed were more statistically significantly that the entire pathway. Second, we compare the significance of the most-perturbed pathways computed by our algorithm to those found by the ActiveModules approach [16]. Third, we compare our results to GSEA [3], a purely gene-set based approach. Fourth, we assess the robustness of our results to the removal of gene expression samples from the input. Finally, we present data in the literature that supports the pathway-cancer connections unearthed by our approach. At this stage, we also compare our results Sub-GSE [7], another gene-set based approach.

### Datasets

We obtained 20 curated pathways from the Netpath database [20]. These pathways include 10 signaling pathways associated with proliferation (Androgen receptor, Alpha6 Beta4 integrin, EGFR1, Hedgehog, ID, Kit receptor, Notch, TGF beta receptor, TNF alpha/NF-kB, and Wnt) and 10 immune response signaling pathways (B cell receptor, T cell receptor, IL-1 IL-2 IL-3 IL-4 IL-5 IL-6 IL-7 and IL-9). We used gene expression measurements in the Global Cancer Map (GCM) [21]. The GCM dataset contains 190 samples spanning 18 cancers (adenocarcinomas of the breast, colon, lung, ovary, pancreas, prostate, and uterus; follicular and large B-cell lymphomas; melanoma; bladder; acute lymphoblastic leukemias of the B cell and T cell; acute myeloid leukemia; renal carcinoma; mesothelioma; and glioblastoma and medulloblastoma, which are two cancers of the central nervous system) and 90 samples from 13 normal tissues (bladder, breast, cerebellum, colon, germinal center, lung, kidney, ovary, pancreas, peripheral blood, prostate, uterus, and whole brain). We compared the samples for each cancer in the dataset to the samples from the corresponding normal tissue (e.g., prostate cancer and normal prostate) using the *t *test. We applied our algorithm to 360 cancer-pathway pairs (18 cancers times 20 pathways). Note that if we performed *k *iterations of permutation testing followed by Benjamini-Hochberg FDR correction, the smallest *p*-value we would obtain would be 360*/k*.

### Significance of partial pathway perturbation

**Figure 1:**
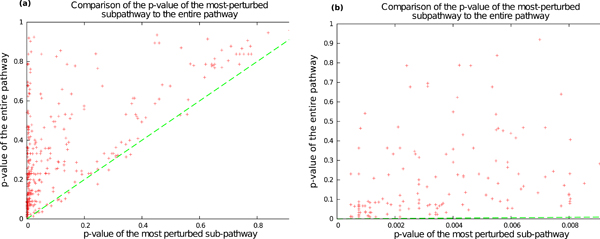
**Results of computing the perturbations of 20 Netpath pathways in 18 human cancers.** Each point represents a pathway-cancer pair: the *x*-axis is the *p*-value of the most-perturbed sub-pathway and the *y*-axis is the *p*-value of the entire pathway in the cancer, with smaller *p*-values indicating greater perturbation. The *x *= *y *line is shown in dashed green. (a) Data for all pathway-cancer pairs. (b) Data restricted to pathway-cancer pairs where the most perturbed sub-pathway has a *p*-value at most 0.01.

When a signaling pathway is perturbed, not all components of the pathway will undergo transcriptional perturbation, because many changes occur at the post-transcriptional or past translational level. Thus when only transcriptional data are available, many pathways may appear to be partially perturbed. An important innovation in our approach is the ability to sensitively detect partial pathway perturbation. To assess the degree that pathways are partially perturbed, for each pathway-cancer pair, we computed the statistical significance of the perturbation score of the most perturbed pathway as well as for the complete pathway. We used 360,000 iterations of the permutation test, thus potentially obtaining *p*-values as low as 0.001. We observed that for 153 pathway-cancer pairs, the most perturbed sub-pathway was significant at the 0.01 level, whereas only 17 pairs were significant at the 0.01 level for complete pathways. In Figure 1, for each of the 360 pathway-cancer pairs, we plot the *p*-value measuring the perturbation of the entire pathway in the cancer (*y*-axis) against the *p*-value of the most-perturbed sub-pathway in that cancer (*x*-axis). Figure 1(a) shows the data for all pathway-cancer pairs, while Figure 1(b) restricts the comparison to those pairs where the most perturbed pathway has a *p*-value at most 0.01. Nearly all points in the plot lie above the green *x *= *y *line. This feature is especially pronounced in Figure 1(b), where *x *= *y *line is just visible above the *x*-axis. Note that the two *x *axes in this figure have ranges differing by two orders of magnitude. For all the pathway-cancer pairs plotted in Figure 1(b), we computed the ratio of the *p*-value of the full pathway to the *p*-value of the most-perturbed pathway. The median value in the distribution of these ratios was 47. Taken together, these results clearly demonstrate that calculating perturbation at the sub-pathway level is substantially more sensitive than calculating it at the whole pathway level.

The pathways in Netpath are carefully curated and we consider them canonical for the purposes of this study. Given the results just presented, a natural question that arises is whether the most-perturbed sub-pathway of a pathway *P *contains a significant fraction of the interactions in *P*. For each pathway, we counted how many interactions appeared to be perturbed in at least one cancer (considering only *p*-values at most 0.01). In other words, for each pathway, we computed the union of its most-perturbed sub-pathways over all the cancers and counted the number of interactions in this union. Table 1 shows that in all but four pathways, fewer than 70% of the interactions in a pathway are perturbed. These data suggest that even such well-studied pathways are perturbed only partially in cancers, at least when taking only gene expression data as an indicator of perturbation. Note that six pathways do not appear in this table because they were not perturbed in any of the cancers. We return to these perturbation results in the section "GCM-Netpath Pathway Perturbations".

**Table 1:** Fraction of interactions in a pathway that occur in any significant most-perturbed sub-pathway

Pathway	#cancers perturbed in	#measured interactions	#perturbed interactions	%perturbed interactions
Alpha6 Beta4 Integrin	14	32	24	75
B Cell Receptor	13	105	79	75.2
EGFR1	16	78	45	57.7
ID	1	53	15	28.3
IL-2	17	75	53	70.7
IL-3	18	58	38	65.5
IL-4	11	36	25	69.4
IL-6	3	46	23	50
IL-7	4	20	12	60.0
Kit Receptor	12	55	29	52.7
T Cell Receptor	12	102	67	65.7
TGF-beta Receptor	15	155	87	56.1
TNF-alpha	17	109	82	75.2

The third column contains the number of interactions that connect genes that are also present in the gene expression data.

### Comparison to ActiveModules

Methods that identify networks that are significantly perturbed in response to a single condition have been developed by multiple groups [16,33,19,18]. Among the approaches that estimate the *p*-value of the differential expression of each gene and use the *p*-values as node weights in a protein interaction network, the ActiveModules algorithm developed by Ideker et al. [16] is widely used and readily available as a plugin for the Cytoscape software package [34]. ActiveModules operates on the same types of data as our approach. ActiveModules defines the score of a subnetwork *Q*(*G'*, *I'*) as:

$$z_{AM}\left(Q\right)=\frac{\sum_{g\in G^{\prime }}z\left(g\right)}{\sqrt{\left|G^{\prime }\right|}}.$$

Note that the set of interactions *I' *does not play a role in the definition of *z_AM _*(*Q*). ActiveModules utilizes the interactions in the network during the search for a subgraph *Q *with the highest value of *z_AM _*(*Q*), by ensuring that *Q *is connected. Thus, interactions play an indirect role in this approach. In contrast, our approach directly incorporates interaction structure into the scoring function.

**Figure 2:**
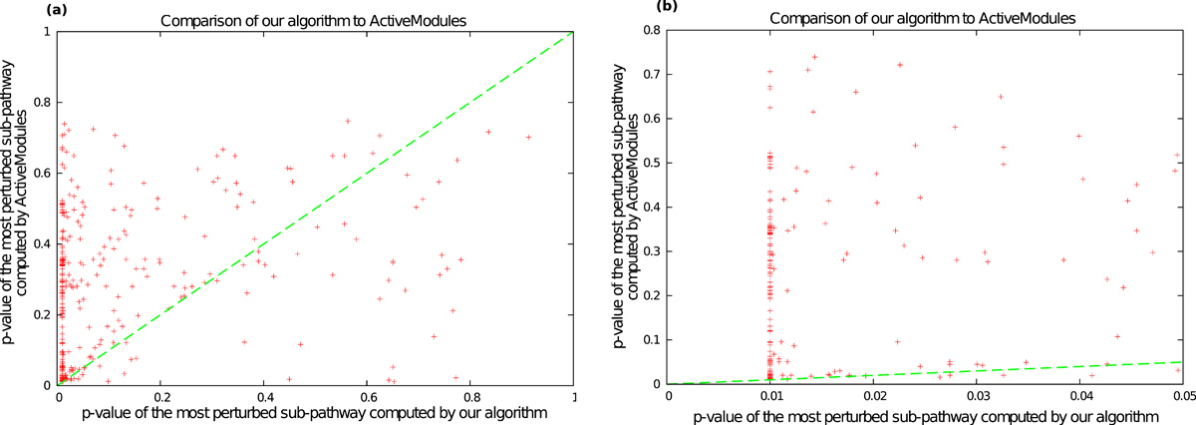
**A comparison of the *p*-values of the most-perturbed sub-pathways computed by ActiveModules to those computed by our algorithm.** Each point represents a pathway-cancer pair: the *x*-axis is the *p*-value of the most-perturbed sub-pathway computed by our algorithm, while the *y*-axis is the *p*-value of the most-perturbed pathway, as computed by ActiveModules. The *x *= *y *line is shown in dashed green. (a) Data for all pathway-cancer pairs. (b) Data restricted to pathway-cancer pairs where the most perturbed sub-pathway computed by our algorithm has a *p*-value at most 0.05.

We compared the significance of the sub-pathways found using our approach to those found using ActiveModules. To estimate *p*-values, we executed both methods on 36,000 randomized pathway datasets. Note that ActiveModules can be computationally intensive to run since it includes an expensive check for sub-network connectivity. Therefore, we performed only 36,000 runs of permutation testing, as opposed to the 360,000 iterations we executed in the section "Significance of Partial Pathway Perturbation". Thus, the smallest *p*-value we could obtain was 0.01. We compared the *p*-values produced by the two algorithms using a method similar to the earlier comparison of most-perturbed pathways to full pathways. In Figure 2(a), each point represents a pathway-cancer pair: the *x*-axis is the *p*-value computed using our approach and the *y*-axis is the *p*-value of the network computed using ActiveModules, with smaller *p*-values indicating greater sensitivity. In Figure 2(b), we only plot these points when our algorithm yields a *p*-value at most 0.05. Note that we used a cutoff of 0.05 instead of 0.01 because the smallest *p*-value we could have obtained in this analysis was 0.01. We chose 0.05 so that we could visualize the range of *p*-values between 0.01 and 0.05. Our algorithm produces a *p*-value less than or equal to ActiveModules for all but 7 of the 232 pathway-cancer pairs that meet this cut-off. Note that in Figure 2(b), the range of *p*-values produced by our algorithm is between 0 and 0.05 whereas the *p*-values computed by ActiveModules span a much wider range. For each pathway-cancer pair in Figure 2(b), we computed the ratio of the *p*-value computed by ActiveModules to the *p*-value estimated by our algorithm. The median value in the distribution of these ratios was 16.5, implying that our algorithm yields *p*-values that are an order of magnitude smaller than ActiveModules, on average. Taken together, these results demonstrate the superior sensitivity of explicitly incorporating interaction structure into scoring sub-pathways.

### Comparison to GSEA

Our approach explicitly uses the interaction structure of pathways to calculate their perturbation. To assess the advantages of this approach, we compared our method to the gene-oriented method GSEA [3]. GSEA compares two phenotypes of interest by sorting all the genes based on the difference in their expression profiles in the two phenotypes, e.g., by using the *t *statistic. Given a gene set of interest, GSEA uses a modified Kolmogorov-Smirnov statistic to test whether the genes in the gene set are ranked toward the top or the bottom of the sorted list. GSEA measures the statistical significance of an observed score by repeatedly permuting the phenotype labels of the samples.

We converted each Netpath pathway into the set of genes that are members of the pathway. We tested each of the 360 pathway-cancer using GSEA, ranking genes by the *t *statistic and generating 100,000 random permutations to assess the statistical significance of the computed scores. GSEA identified no Netpath gene set as significant in any cancer, even with an FDR-adjusted *p*-value less than 0.1. We had observed that perturbed pathways computed by our method may contain both up- and down-regulated genes. We reasoned that GSEA may not detect corresponding gene sets as significantly differentially expressed since these gene sets contain both genes with low ranks (large positive *t *statistics) and with high ranks (large negative *t *statistics). Therefore, we repeated the analysis using GSEA's option to rank genes by the absolute value of the *t *statistic. Even with this option, GSEA identified no pathway-cancer pairs as significant, even at the 0.1 level.

GSEA uses the null hypothesis that the distribution of the perturbation of the genes in a particular gene set is the same as the distribution of the rest of the genes measured in the transcriptional data set. Our approach uses the null hypothesis that the distribution of the perturbation of the genes in a particular pathway *P *is the same as the distribution of an equal number of randomly-selected genes, where the interactions between the randomly-selected genes are isomorphic to the interactions in *P*. To test the possibility that the stricter null hypothesis of GSEA prevents it from finding significant perturbations detected by our method, we used our results to construct a new gene set for each cancer. Each new gene set was composed of only those genes that participate in at least one of the most-perturbed sub-pathways in that cancer as determined by our method. We applied GSEA to these new gene sets, ranking genes by the absolute value of the *t *statistic. For 13 out of the 18 cancers, GSEA found that the combined gene set constructed based on our results was more significant than the gene set for any individual pathway. Yet, only two of these combined gene sets had an FDR-corrected *p*-value less than 0.1. From this comparison with GSEA, we conclude that incorporating interaction structure is an important aspect of determining pathway perturbation.

### Robustness of our approach to missing data

We evaluated the robustness of our approach to missing data. The GCM data contains multiple samples for each cancer. For each pathway-cancer pair, we removed each sample for that cancer from the input and re-computed the most perturbed sub-pathway and its statistical significance. This process was computationally intensive since we had to compute the statistical significance for each pathway-cancer about 15 times (depending on the number of samples in each cancer). Therefore, we ran 36,000 iterations of permutation testing, yielding *p*-values at least as large as 0.01. For each pathway-cancer pair, we counted how many leave-one-out datasets yielded results that were similar to results obtained with the complete dataset. Specifically, if the pair was statistically significant, i.e., had a *p*-value at most 0.05 in the full dataset, we counted the fraction of leave-one-out datasets for which the most-perturbed sub-pathway also had a *p*-value at most 0.05. Conversely, for pathway-cancer pairs that were not statistically significant, i.e., had a *p*-value greater than 0.05, we counted the fraction of leave-one-out datasets for which the most perturbed sub-pathway also had a *p*-value greater than 0.05. We expected all these fractions to be close to 1, i.e., the significance for the full dataset would hold in the leave-one-out dataset as well.

**Figure 3:**
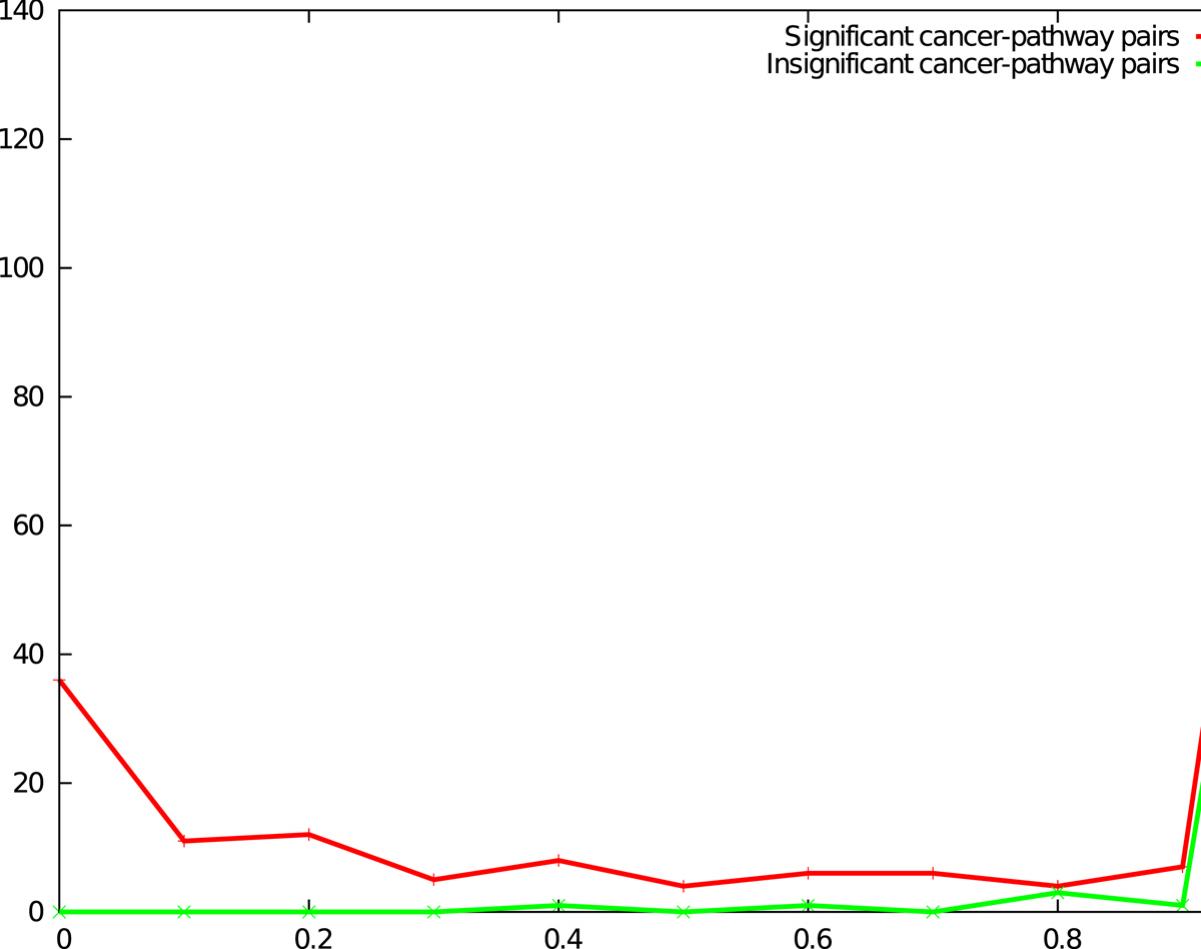
**Results of robustness analysis.** The *x*-axis plots the fraction of leave-one-out datasets for which a cancer-pathway pair was significant at the 0.05 level (for pairs that were significant with the full dataset, red curve) or not significant (for pairs that were not significant with the full dataset, green curve). The *y*-axis plots the number of cancer-pathway pairs that were significant (red curve) or not significant (green curve).

Of the 360 cancer-pathway pairs, 238 pairs were significant, i.e., they had a *p*-value at most 0.05 with the full dataset, leaving 122 pairs with a *p*-value greater than 0.05. As shown in Figure 3, of the 238 significant cancer-pathway pairs, 58% of pairs (138 pairs) had a robustness of 1, i.e., every time we removed one of the samples for that cancer, the cancer-pathway pair had a *p*-value at most 0.05 with the remaining samples. Only 32% of the 258 pairs failed the significance test for more than half the samples. For the 122 pairs that were insignificant, as many as 96% (117 pairs) had a robustness of 1, i.e., the removal of every sample kept the perturbation *p*-value larger than 0.05. We obtained very similar trends if we performed this analysis with a *p*-value threshold of 0.01. These results suggest that our method is highly robust to modest changes in the input gene expression data.

### GCM-Netpath pathway perturbations

**Figure 4:**
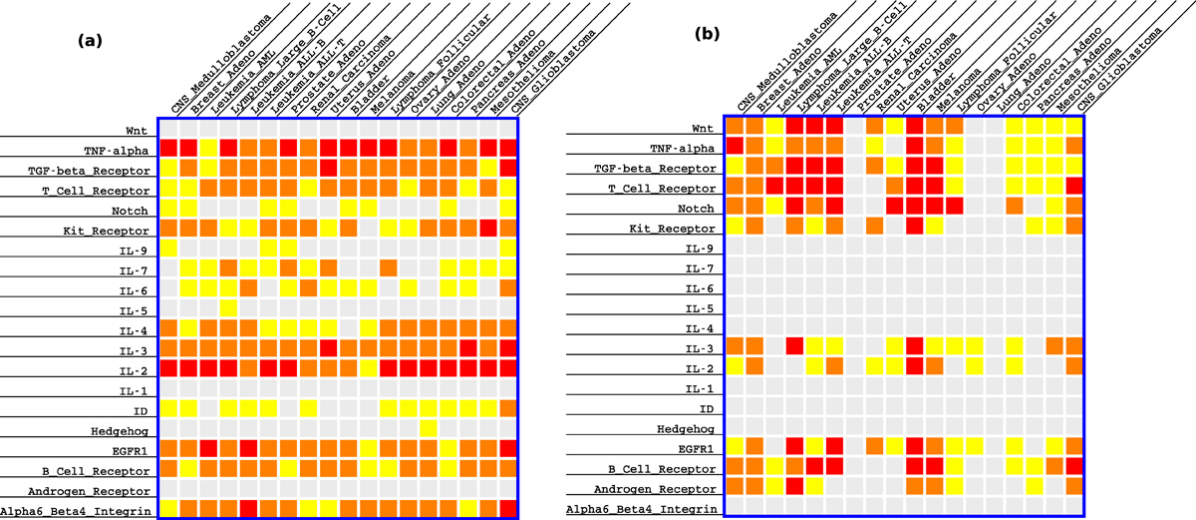
**An overview of the perturbations of 20 Netpath pathway in 18 cancers in the GCM dataset.** Each row is a pathway and each column is a cancer. The color of a cell indicates the FDR-corrected *p*-value of the perturbation of a pathway in a cancer: red = 0.001, orange *≤ *0.01, yellow *≤ *0.05, and gray *>*0.05. (a) Results obtained by our algorithm. (b) Results obtained with Sub-GSE.

We assembled the results obtained in the section "Significance of partial pathway perturbation" on the differential perturbation of each Netpath pathway in each cancer in the GCM into the matrix shown in Figure 4(a). Of the 360 pathway-cancer pairs we analyzed, 35 pairs had FDR-corrected *p*-values equal to 0.001, 118 pairs had *p*-values greater than 0.001 and at most 0.01 and 78 pairs had *p*-values greater than 0.01 and at most 0.05. Recall that we used using 360,000 permutations to obtain these results. Therefore, we could obtain *p*-values as low as 0.001. Many pathways were perturbed in almost all the cancers, with *p*-value less than 0.01: IL-3 pathway (18), IL-2 pathway (17), TNF-alpha Pathway (17) EGFR1 pathway (16), TGF-beta receptor pathway (15), Alpha6 Beta4 Integrin pathway (14), and B Cell Receptor pathway (13). Seven pathways, including the Androgen receptor, Hedgehog, IL-1, IL-5, IL-9, Notch, and Wnt signaling pathways were not significantly perturbed by any condition in our dataset, leaving 13 pathways that were perturbed by at least one cancer. Many cancers perturb more than half of these 13 pathways. A complete analysis of these results is beyond the scope of this paper. We focus on literature support for our results on two important pathways: tumor necrosis factor alpha (TNF-alpha) and interleukin 2 (IL-2). Both pathways are associated with many tumors (11 and 13, respectively) in our results. Support for these associations can be found in literature.

Both of these pathways have down-regulated expression in multiple tumor types [35,36]. The TNF-alpha pathway is perturbed in association with CNS, melanoma, and bladder tumors, among others. TNF-alpha is down regulated in tumors like melanoma [35]. Studies have observed the cytotoxic effects of TNF-alpha on medulloblastoma [37]. Other work has found that TNF-alpha is an important factor in breast cancer promotion and survival [38]. TNF is used for localized treatment of metastatic melanomas and other irresectable tumors [39]. Recombinant TNF has been effectively used to treat bladder tumors *in vivo *[40]. The interleukin 2 (IL2) pathway is another pathway that we find perturbed by many cancers. The IL2 pathway is an immune signaling pathway that is commonly down regulated in tumors like T-cell lymphoma [36]. Like TNF-alpha, IL2 is also added exogenously to treat multiple cancer types including metastatic melanoma [41] and superficial bladder tumors [42]. These treatments were found to work in breast cancer cell lines that express the interleukin 2 receptor on the cell surface [43].

A comprehensive understanding of pathway perturbations has important implications in disease treatment. As noted above, exogenous treatment with recombinant TNF and IL2 have had success in mitigating tumor progression in a number of diseases [37,39,40,42]. The success of these treatments illustrates that reversing pathway perturbation to a pre-cancerous state can help to restore the healthy phenotype. Therefore, it is important to characterize both the extent and direction of pathway perturbation across diseases.

### Comparison to Sub-GSE

Sub-GSE [7] is another gene-set oriented method that has been reported to be more sensitive than GSEA. Therefore, we ran Sub-GSE on the GCM and Netpath data and compared the results to our perturbed pathways-cancer pairs. We ran Sub-GSE with 10,000 iterations (for the permutation test Sub-GSE uses to compute significance). The Sub-GSE software gave memory allocation errors for approximately 20,000 or more iterations. Since we could not run Sub-GSE for larger numbers of iterations, we did not correct the *p*-values yielded by Sub-GSE for multiple hypotheses testing.

We found that both Sub-GSE and our method identified many common pathway-cancer associations (Figure 4). However, Sub-GSE failed to identify any cancer associations for the ID or the alpha 6 beta 4 integrin signaling pathways. These pathways are known to be perturbed in multiple tumor types. The ID signaling pathway has been associated with carcinogenesis by supporting tumor cell migration and invasion [44]. Although the ID pathway is mostly dormant after embryogenesis, the pathway is reactivated during tumor progression [45]. Upregulation of the alpha 6 beta 4 integrin pathway has been associated with metastatic potential in many cancers [46]. In tumor microenvironments, alpha 6 beta 4 is re-localized to the leading edge of tumor cells and promotes invasion [46]. While Sub-GSE has superior sensitivity to GSEA, Sub-GSE was not sensitive enough to identify these important associations.

However, our approach was not able to identify significant associations with the Wnt or Androgen receptor pathways that were detected by Sub-GSE. Both pathways are known to be associated with multiple cancer types. Disregulation of the Wnt signaling pathway leads to upregulated expression of B-catenin, which ultimately results in increased proliferation of tumor cells [47]. Sub-GSE is able to identify numerous cancers in which the Androgen receptor pathway is perturbed. However, neither Sub-GSE nor our method was detect the well-known and widely studied association between this pathway and prostate cancer [48]. We note that the increased sensitivity of Sub-GSE in the case of these two pathways may arise from the fact that we did not adjust for testing multiple hypotheses in the case of Sub-GSE.

## Summary

Our results indicate that integrating differential gene expression with the interaction structure in a pathway is a powerful approach for detecting links between a cancer and the pathways perturbed in it. The use of Stouffer's z-score to combine multiple *p*-values provides an important advantage over methods that consider pathway membership alone: in many perturbed pathways, we noticed that the receptor protein at the head of the pathway was very slightly differentially expressed, often not to a statistically significant extent, whereas many genes with products downstream of the receptor were differentially expressed (data not shown). Our use of meta analysis to combine *p*-values enabled detection of the perturbation of the pathway even in such cases.

There are several avenues for future work. Our method currently ignores the direction of differential expression of each gene. Incorporating this information is important. It is also essential to take into account prior information on whether any interactions are regulatory and on the type of regulation implied by an interaction [10]. Such information may yield pathways with both directed and undirected interactions. Finally, it would be interesting to use universal protein interaction networks in order to expand curated pathways.

## Competing interests

The authors declare that they have no competing interests.

## Authors' contributions

All authors developed the method, and CGR implemented the algorithm. CGR and TMM analyzed the results, and all authors wrote the article.
